# The supraspinatus fascia: a consistently two-layered structure with nociceptive innervation and differential functional anatomy

**DOI:** 10.1007/s00276-026-03949-0

**Published:** 2026-07-28

**Authors:** Lara Herrmann, Charlotte Kulow, Pierre Hepp, Hanno Steinke

**Affiliations:** 1 Institute for Anatomy, Leipzig University, Liebigstraße 13, 04103 Leipzig, Germany; 2 Department of Orthopaedics and Trauma Surgery, University Hospital Leipzig, Liebigstraße 20, 04103 Leipzig, Germany

**Keywords:** supraspinatus fascia, shoulder fascia, scapular pain, rotator cuff tears, myofascial pain, nerve entrapment syndrome

## Abstract

**Purpose:**

Pain in the scapular region is a common musculoskeletal complaint, yet its anatomical basis often remains unclear. This study investigates the supraspinatus fascia as a potential contributor to shoulder pain, with particular emphasis on its surgical relevance.

**Method:**

A total of 24 body donors were examined using dissection and histological analysis to characterize the fascia’s morphology, topography, and neurovascular features.

**Results:**

The supraspinatus fascia consistently demonstrated a distinct two-layered architecture. Both layers showed considerable interindividual variability, ranging from thin and translucent to dense and collagen rich. There is an interposed fat layer between the superficial and deep fascial layer suggesting a potential sliding interface. The fascia forms a tubular sheath around the supraspinatus muscle, attaching to the margins of the supraspinatus fossa as well as the superior transverse scapular ligament, clavicle, and coracoid process. The deep layer is continuous with the subacromial bursa and the posterior origin of the supraspinatus muscle. In contrast, the superficial layer integrates into surrounding fascial systems, including connections to the fasciae of the levator scapulae and rhomboid minor muscles. Histological analysis identified neurovascular bundles within and traversing both fascial layers. A subset of nerve fibres showed substance P immunoreactivity, supporting a nociceptive role of the supraspinatus fascia.

**Conclusion:**

The supraspinatus fascia is a complex structure rather than a passive wrapping. Its distinct two-layered architecture and substance P-positive innervation provide an anatomical basis for idiopathic scapular pain. Clinically, the deep layer’s continuity with the subacromial bursa suggests that inadequate fascial release during subacromial decompression may lead to persistent symptoms. Finally, the superficial layer’s connection to the “levator-rhomboid-minor-fascia” establishes a myofascial bridge for force transmission, suggesting that “shoulder pain” could be a manifestation of cervicoscapular fascial tension.

## Introduction

Shoulder pain is one of the most common everyday complaints of today’s adult population, yet its aetiology often remains unclear despite extensive clinical investigation of muscular, neural, and osseous structures. While traditional diagnostic focus has centred on rotator cuff pathology, bursitis, and nerve entrapment, these factors alone do not fully explain the persistence of pain in a substantial proportion of patients.

Although shoulder fasciae have already been described in anatomical atlases several centuries ago, more recent research has been directed toward fascial tissues as potential contributors to musculoskeletal function and pain. Fascia is now recognised as a continuous three-dimensional connective tissue network that plays a significant role in force transmission and structural integrity [16]. Fascia has also been shown to contain a rich supply of free nerve endings and mechanoreceptors, supporting the concept that fascia may also act as a nociceptive and sensory organ [14, 17, 21].

Anatomical investigations further indicate that fascia is not merely a passive envelope, but part of the musculoskeletal apparatus, contributing actively to movement and stability [8, 22, 23]. These findings suggest that fascial structures may be involved in the pathophysiology of pain syndromes, including those affecting the shoulder girdle.

Within the rotator cuff, the supraspinatus muscle is enclosed by a strong fibrous sheath, the supraspinatus fascia. Although the fascia was mentioned as early as 1830 [19], a detailed description and investigation of its structure, characteristics, and function has interestingly not yet been provided. We, therefore, aimed to provide an anatomical description of the supraspinatus fascia, assess its variability, and explore its potential implications for shoulder 

function and pain. By integrating macroscopic dissection with histological analysis, we sought to address the gap in knowledge regarding fascial tissue organisation within the supraspinatus region, and to highlight why fascial structures, despite growing evidence of their functional significance, remain underemphasised in both anatomical research and clinical diagnostics.

## Methods

For our research, we examined 24 body-donors, aged between 66 and 96, 11 males and 13 females. Replacement shoulders or shoulders with obvious surgical intervention were not included in the study. All tissue samples were obtained from body donors who had previously given informed consent for the use of their bodies for research and educational purposes post-mortally. Institutional approval was obtained in accordance with the Saxonian Death and Funeral Act of 1994. Signed body donor consent forms are available on reasonable request from the corresponding author.

### Dissection on fresh specimens

To examine the supraspinatus fascia, we used 24 fresh shoulder specimens that had already been removed and frozen earlier. Sixteen specimens were dissected using the same protocol to describe the anatomical morphology of the supraspinatus fascia. Fascial thickness measurements were obtained in 11 of these 16 specimens. An additional eight specimens were used for histological examination, photography, and further assessment of fascial morphology (Table 1).

**Table 1:** Demographic characteristics of the body donors: including case number, age, sex, side of the shoulder examined, and the respective use of each specimen for morphological dissection, histological analysis, and documentation

Case-nummer	Age	Sex	Used shoulder	Utilization
1a	95	Female	Left	Dissection, measurement
1b	95	Female	Right	Dissection, measurement
2a	96	Female	Left	Dissection
2b	96	Female	Right	Dissection, measurement
3b	96	Female	Right	Dissection
4a	91	Male	Left	Dissection
4b	91	Male	Right	Dissection, measurement
5a	77	Male	Left	Dissection, Fig. 2b
5b	77	Male	Right	Dissection
6b	85	Male	Right	Dissection, measurement
7a	90	Female	Left	Dissection
8b	67	Female	Right	Dissection, measurement
9a	70	Female	Left	Dissection, measurement
10a	82	Male	Left	Dissection, measurement, Fig. 4a
11b	86	Female	Right	Dissection, measurement
12a	88	Male	Left	Dissection, measurement
12b	88	Male	Right	Dissection, measurement, Fig. 2a
13a	92	Male	Left	Photos, Fig. 2c
14b	89	Male	Right	Histology, Fig. 5
15a	84	Female	Left	Photos, Figs. 2d/1b and c
16a	86	Female	Left	Photos, Fig. 1d
17a	90	Female	Left	Dissection
17b	90	Female	Right	Dissection, Fig. 4b
18a	73	Male	Left	Photos, Fig. 3

Shoulder specimens were obtained from body donors in the prone position. Skin, subcutaneous tissue, and the surrounding musculature were removed as required to expose the shoulder girdle. The clavicle, scapula, and proximal humerus were transected, allowing removal of the shoulder specimen as a single unit. Specimens were wrapped in plastic foil, frozen at − 20 °C, and thawed prior to dissection. During thawing, all specimens remained sealed within the protective foil and were not exposed directly to water.

### Removal of the trapezius muscle and the humeral head

To expose the supraspinatus fascia, the trapezius muscle and its overlying fascia were carefully detached from their insertions on the scapular spine, acromion, and clavicle and reflected superiorly. This revealed a distinct whitish-yellow fascial layer between the trapezius and supraspinatus muscles.

The remaining deltoid muscle was then removed by separating it from the underlying fascia and from its attachments to the clavicle, acromion, and scapular spine. After opening the fascia and the glenohumeral joint capsule, the humeral head was inspected for degenerative changes and subsequently removed.

### Access to the supraspinatus muscle

To preserve the supraspinatus fascia, the supraspinatus muscle was approached from the deep aspect through the supraspinatus fossa. The subscapularis and remaining surrounding muscles were removed, and a small bony window was created in the centre of the supraspinatus fossa using an oscillating saw. The opening was enlarged while preserving the bony margins of the fossa. The supraspinatus muscle was then carefully detached from the fascia until the latter could be visualized from its deep surface while remaining attached to the margins of the fossa.

### Access to the supraspinatus fascia

To completely expose the supraspinatus fascia, we turned the specimen over, so it lay on what once was the base of the supraspinatus fossa. We then searched for the centre of the scapular spine and made a one-millimetre-deep incision above the centre of the scapular spine into the fatty connectives, former between trapezius and supraspinatus muscles. We widened the incision in all directions to reveal the supraspinatus fascia, without damaging it, using blunt, rounded surgical scissors and anatomical forceps. Placing the same scissors between the layers we separated them carefully.

### Visualizing the insertions of the supraspinatus fascia on the acromion, the clavicle and the coracoid process

To visualize the complete course of the supraspinatus fascia, the remaining muscle fibres were carefully separated from the fascia through the previously created approach through the posterior wall of the supraspinatus fossa. The fascia was followed laterally towards its attachments to the subacromial bursa and acromion. Remaining connective tissues attached to the scapula and lateral clavicle were subsequently removed to allow complete visualization of the fascial insertions.

### Measuring the thickness of the supraspinatus fascia

**Fig. 1:**
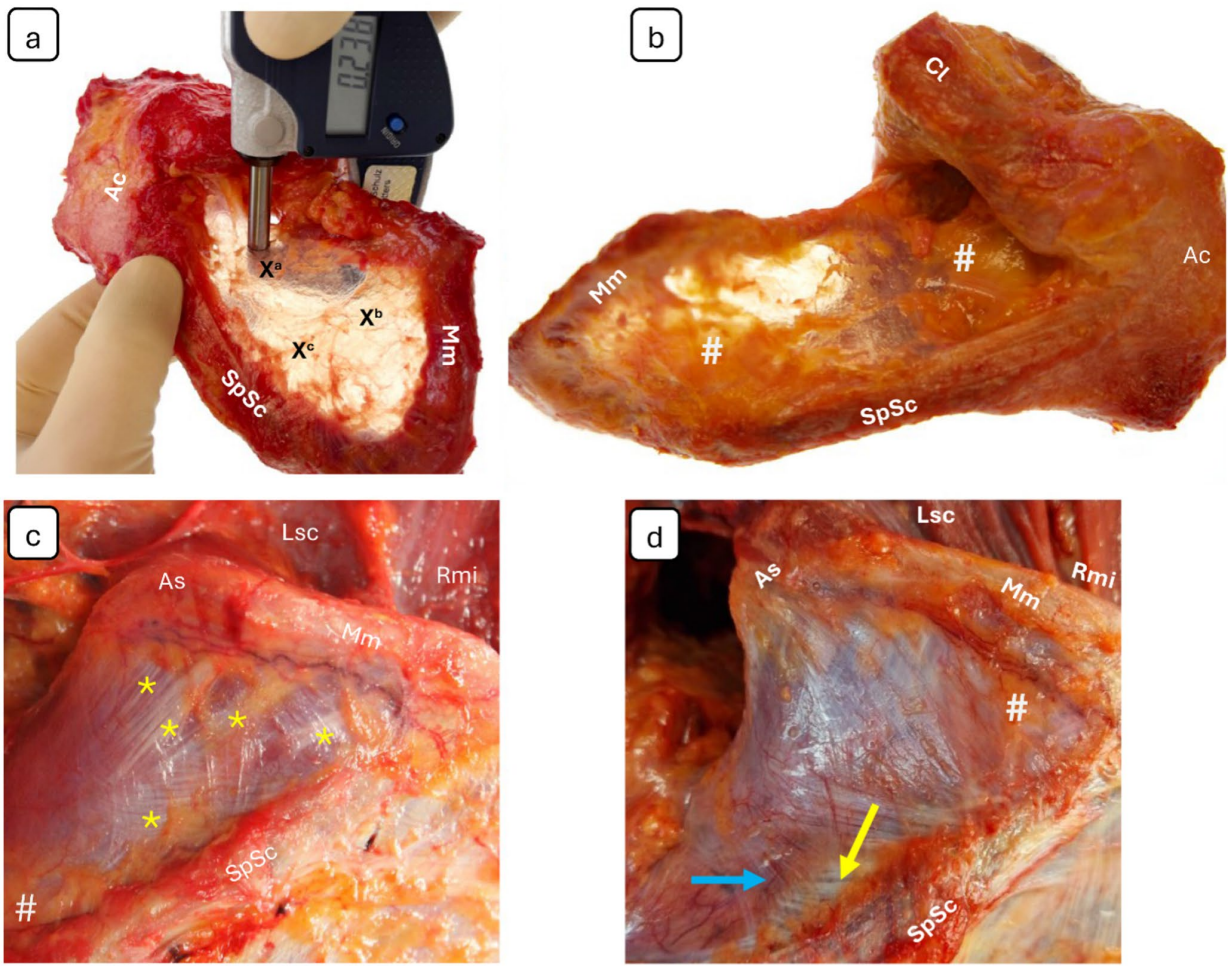
**a** Dorsal view, fresh specimen. After removal of the skin and subcutaneous tissue, the trapezius muscle was reflected cranially. The supraspinatus fascia was observed attaching to the spine of the scapula (SpSc), the medial margin (Mm), and the superior angle of the scapula (As). All muscles attached to the scapula (i.e. trapezius, deltoid, infraspinatus, subscapularis, rhomboid minor and major, levator scapulae, and supraspinatus) were removed, leaving only the supraspinatus fascia attached to its origins at the spine of the scapula (SpSc), the medial margin (Mm), the clavicle (Cl), and the acromion (Ac). This specimen shows little to no visible fibres and appears almost transparent. X^a^ (lateral measuring point), X^b^ (medial measuring point), and X^c^ (measuring point above the centre of the spine of the scapula) mark three measuring points, which were used in every measured specimen. **b** Dorsal view, fresh specimen. Same procedure as Fig. 1a. The white # indicates fatty infiltration located between the **superficial** and **deep** layer of the supraspinatus fascia (also visible in panels and c). **c** Dorsal view, fresh specimen. Same procedure as described in Fig. 1a. but the levator scapulae (Lsc) and rhomboid minor (Rmi) muscles remained attached to the medial margin (Mm) of the scapula. Marked with yellow * the fibres within the fascia can be seen, becoming less strong laterally. **d** Dorsal view, fresh specimen. Same procedure as Fig. 1c. The two distinct fascial layers could be differentiated into the **superficial** layer (blue arrow) and the **deep** layer (yellow arrow) based on the differing orientation of their fibres

On eleven specimens, we defined three measuring points at the beginning of our research, at which we measured the thickness of each fascia using the Mitutoya Digital Micrometre 0–25 measuring device accurate to one micrometre. We defined point one as the midpoint on a vertical line between the suprascapular notch and the spine of the scapula (X^a^). Point two was defined as the intersection of a vertical line from the most medial quarter of the scapular spine with a line from the superior angle of the scapula (X^b^). We defined a third point as a point on a vertical line one-centimetre-long, starting from the centre of the spine of the scapula (X^c^) (Fig. 1a). For each specimen, the mean fascial thickness was calculated by averaging the measurements obtained at the three predefined points. The overall mean fascial thickness was then determined as the mean of these specimen-specific values.

### Histology

Three probes were removed from the fascia at the same points we measured the fascia. They were then fixed in formaldehyde (ROTH Histofix 4%), embedded in paraffin, and then stained with Kernechtrubin-Anilinblau-Orange-Staining (Waldeck GmbH & Co. KG, Münster, Germany. Another probe was removed from the centre of the fascia. This probe was also fixed in formaldehyde (ROTH Histofix 4%), embedded in paraffin, and then likewise stained. We, furthermore, tested that probe for Substance P (Anti-Substance P Antibody, rabbit polyclonal, Merck (Chemicon)). Neurofilament immunoreaction was also performed for this probe (Neurofilament MAB5262, Millipore, Merck, Darmstadt, Germany).

## Results

### Attachments of the supraspinatus fascia

#### Anterior attachments

In 11 out of 16 cases, the supraspinatus fascia attaches to the superior part of the superior transverse scapular ligament. In comparison, in five out of 16 cases, it ran anteriorly-caudally to form a fascial channel around the supraspinatus muscle without attaching to the superior transverse scapular ligament but attaching to the superior border of the scapula.

#### Anterior-lateral

In 14 out of 16 cases, the fascia attaches to the lateral fourth of the caudal surface of the clavicle. The higher the content of visible fibres was on the part of the fascia that covers the supraspinatus fossa, the stronger of a connection was seen to the clavicle.

Along its lateral course to the acromion, 12 out of 16 cases showed fibres running off ventrally and attaching to the neck of the coracoid process.

#### Anterior-cranial

When following the fascia´s course cranially-anteriorly from its attachment to the caudal surface of the clavicle, an attachment to the cranial and dorsal part of the trapezoid and conoid ligaments can be seen in all 16 cases, forming the ventral border of the fascial channel for the supraspinatus muscle. In all dissected specimens, the lateral border is formed by the fascia´s attachment to the medial part of the posterior coracoacromial ligament.

#### Lateral attachments

The fascia follows the supraspinatus muscle from medial to lateral. It forms a channel for the muscle below the acromion in all cases (Fig. 3), attaching to the visceral layer of the subacromial bursa and finally to the caudal surface of the acromion within 0.5–2 cm of its lateral end. In all cases, fibres of the fascia also extended to the cranial surface of the subacromial bursa before attaching to the upper part of the glenohumeral capsule.

#### Dorsal attachments

Even though the fascia follows the supraspinatus muscle along its course below the acromion, it does not surround the muscle in its entire course. In the supraspinatus fossa, the fascia serves as the dorsal boundary of the bony fossa and thus attaches to its bony borders. It, therefore, attaches to the superior angle, the medial and superior border of the scapula, and the spine of the scapula in all 16 cases (Fig. 1b). At their attachment to the spine of the scapula, fibres of the fascia also interweave with those of the infraspinatus fascia.

#### Attachments to the supraspinatus muscle

**Fig. 2:**
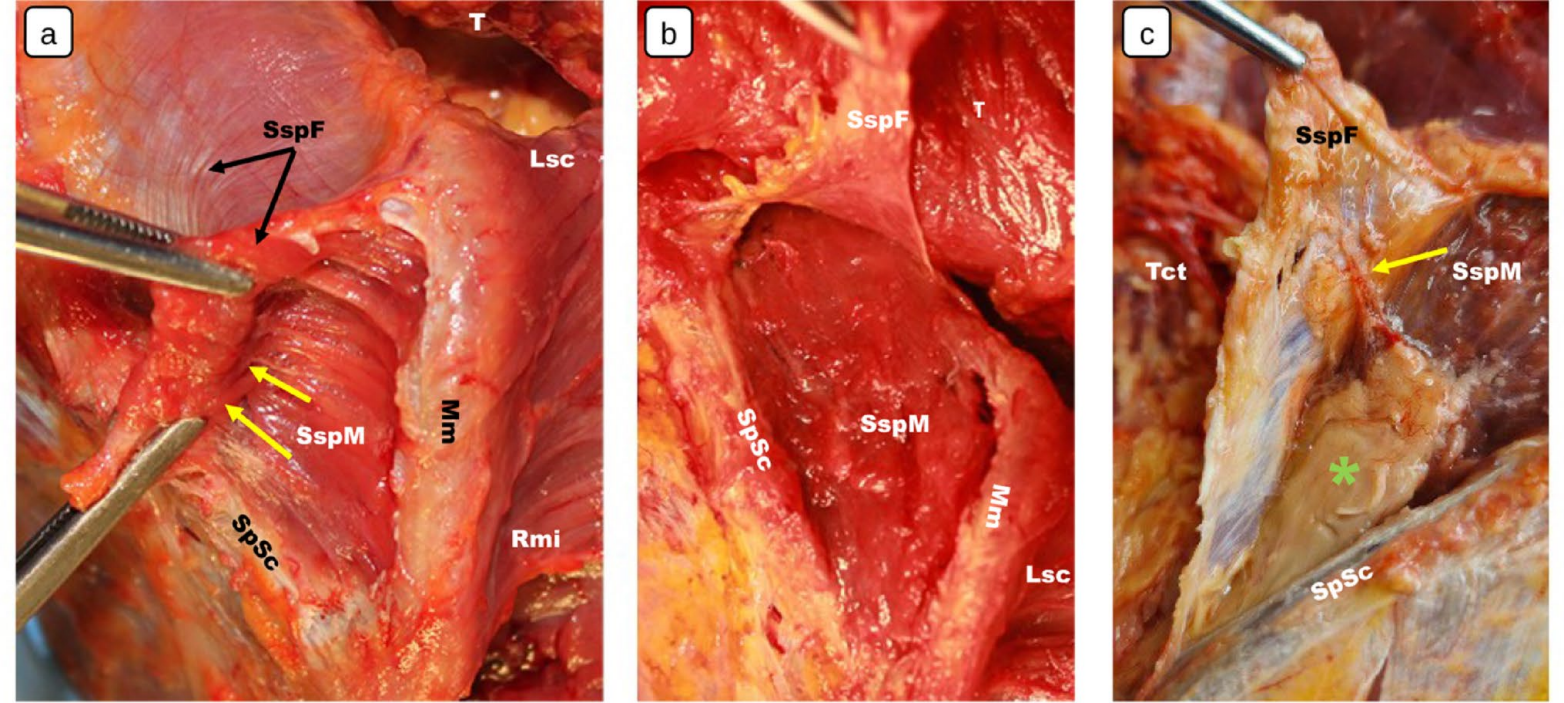
** a** Dorsal view, fresh specimen. After removal of skin and subcutaneous tissue, the trapezius (T) was reflected cranially; levator scapulae (Lsc) and rhomboid minor (Rmi) remained attached to the medial margin (Mm). The supraspinatus fascia (Sspf), attached to the spine (SpSc) and medial margin (Mm), was detached from these origins and reflected craniolaterally. The yellow arrows indicate the attachment of the posterior portion of the supraspinatus muscle (SspM) to the medial part of the supraspinatus fascia (Sspf) **b** Dorsal view, fresh specimen. Preparation as in (Fig. 2a). After detachment from the spine (SpSc) and medial margin (Mm) and craniolateral reflection, the lateral supraspinatus fascia (Sspf) showed no attachment to the underlying supraspinatus muscle (SspM) (same specimen as in Fig. 2a). **c** Dorsal view, fresh specimen. After removal of skin and subcutaneous tissue, the trapezius muscle and underlying connective tissue (Tct) were reflected cranially. The supraspinatus fascia (Sspf) was detached from the spine of the scapula (SpSc) and medial margin (Mm) and reflected cranially. The yellow arrow indicates a supraspinatus muscle (SspM) fibre attaching to the fascia; green * marks fatty infiltration without fascial attachment

The supraspinatus fascia is attached to the supraspinatus muscle in all dissected specimens (Fig. 2a). The posterior portion of the muscle originates from the scapular spine as well as the medial third to half of the supraspinatus fascia. In the nine out of 16 specimens that showed prominent fibres, the attachment of the medial third of the fascia to the supraspinatus muscle was more pronounced than in those with less apparent fibres, although the muscles origin from the fascia was still consistently present. Especially on the caudal surface of the visible fascia fibres, the strength of the adhesion between the muscle and the fascia could be seen since removing the muscle from the caudal surface of the fibres of the medial part of the fascia was completely impossible without damaging the fascia (Fig. 2a).

In contrast, detaching the muscle from the lateral parts of the supraspinatus fascia was straightforward because the adhesion between the muscle and the fascia progressively decreased laterally (Fig. 2b). The adhesion to the medial part of the visible fascia covering the supraspinatus fossa was noticeably less strong in four out of 16 cases where less visible fibres could be found within the fascia. In these four cases, fascial attachments to the muscle were evident on the medial fifth of the visible fascia covering the supraspinatus fossa, and almost no adhesion following the fasciae´s courses laterally. On the other hand, nine out of 16 cases showed strong visible fibres and thus strong attachments between the muscle and the fascia in the complete medial third of the fascia.

### Fibre content and thickness of the fascia

#### Fibre content

When examining the fascia from medial to lateral, apparent differences in the appearance of the fascia can be recognised (Figs. 1a, c and d and 4a). While the medial parts of the fascia covering the supraspinatus fossa appeared clear to whitish and fibre-rich, depending on the fibre content, the fascia appears to become more macroscopically delicate laterally (Fig. 1c). This is not least due to the fibres, which are already clearly visible macroscopically and can only be seen in the area of the fascia covering the supraspinatus fossa (Fig. 1c). All examined specimens showed macroscopic fibres to varying degrees. In eleven of 14 specimens the visible fibres of the fascia were so prominent that they were even palpable. All specimens showed the most significant presence of visible fibres at the attachment of the fascia on the medial two-thirds of the spine of the scapula which was characterized by a higher density of visible fibres and increased fascial thickness in this region of the fascia (Table 2).

**Table 2:** Measurements: Measurements were taken at the intersection of a vertical line from the most medial quarter of the scapular spine with a line from the superior angle of the scapula (X^b^), the midpoint on a vertical line between the suprascapular notch and the spine of the scapula (X^a^) and a point on a vertical line one-centimetre-long, starting from the centre of the spine of the scapula (X^c^)

Case number	Thickness lateral (X^a^)	Thickness medial (X^b^)	Thickness above spine of the scapula (X^c^)	Fibre content
1 a	0.238 mm	0.107 mm	0.493 mm	Low
1 b	0.149 mm	0.063 mm	0.193 mm	Low
2 b	0.217 mm	0.092 mm	0.144 mm	High
4 b	0.133 mm	0.293 mm	0.641 mm	High
6 b	0.101 mm	0.117 mm	0.171 mm	High
8 b	0.143 mm	0.105 mm	1.840 mm	Minimal
9 a	0.121 mm	0.137 mm	0.167 mm	Minimal
10 a	0.156 mm	0.217 mm	0.444 mm	Very high
11 b	0.181 mm	0.052 mm	0.254 mm	High
12 a	0.150 mm	0.157 mm	0.260 mm	High
12 b	0.220 mm	0.270 mm	0.522 mm	High
	Arithmetic mean: 0.222 mm	Arithmetic mean: 0.164 mm	Arithmetic mean: 0.466 mm	

To determine the mean fascial thickness, we first calculated the arithmetic mean of the measurements obtained at each of the three measurement points. The overall mean fascial thickness was then calculated as the average of the three resulting values

#### Thickness

In all 11 specimens whose thicknesses were measured, the fascia was thickest just above its attachment to the spine of the scapula, which varied between 0.722 and 0.144 mm. In five out of six specimens, the fascia was thicker at the lateral measuring point than at the medial, with values ranging from 0.101 to 0.238 mm. In the other six specimens, the fascia was thicker at the medial measuring point, and the measured values were between 0.052 and 0.293 mm (Fig. 1a).

### Additional observations

#### Layers of the fascia

When examining the supraspinatus fascia macroscopically, it became clear that the supraspinatus fascia consists of at least two layers (Fig. 1d) which are closely apposed but can be displaced relative to one another. The multilayered structure of the fascia was further evidenced by the organisation of visible fibres, which displayed differing directional alignments across the superimposed layers (Figs. 1a, c and d and 4a). Between these layers, 14 out of 16 cases showed at least a little fatty infiltration (Figs. 1b and c and 2c) on the lateral part of the fascia shortly before it runs below the acromion, which extends anteriorly in the direction of the clavicle. In all dissected specimens, the multilayered architecture of the supraspinatus fascia was evident due to the differing orientations of the fascial fibres. Furthermore, the individual layers could consistently be separated and displaced relative to one another, allowing them to be distinguished macroscopically. The **deep** fascial layer could also be differentiated from the epimysium of the supraspinatus muscle. This distinction became particularly evident in specimens with fatty infiltration between the fascia and the muscle, where the **deep** fascial layer was located superficial to the fatty tissue, whereas the muscle together with its epimysium was situated deep to it. However, the multilayered fascial architecture and the separability of the layers were observed consistently, irrespective of the presence or absence of fatty infiltration.

#### Connection and fatty infiltrate between the supraspinatus fascia and the fascia below the trapezius muscle

In five out of 16 cases, the initial differentiation between the supraspinatus fascia and the fascia below the trapezius muscle was challenging at first glance because the “subtrapezius fascia” lies directly on top of the supraspinatus fascia. In these specimens, however, these two fasciae are not structurally fused but form a gliding space between the trapezius and the supraspinatus muscle so that they can easily be separated by entering the gliding space between the two fasciae. This gliding space, however, is filled with a fatty infiltrate in most cases (eleven out of 16), which then separates the two fasciae. Depending on the extent of the fatty infiltrate, it fills in the entire gliding space between the two fasciae or just a part of it. In all specimens that showed a fatty infiltrate between the two fasciae, a higher fat content was evident laterally than medially.

Following the fascia laterally and anteriorly in the direction of the clavicle, the increase of fatty infiltrate was recognizable in all dissected specimens, even in the ones that did not show any fat in between the subtrapezius and supraspinatus fascia.

**Fig. 3:**
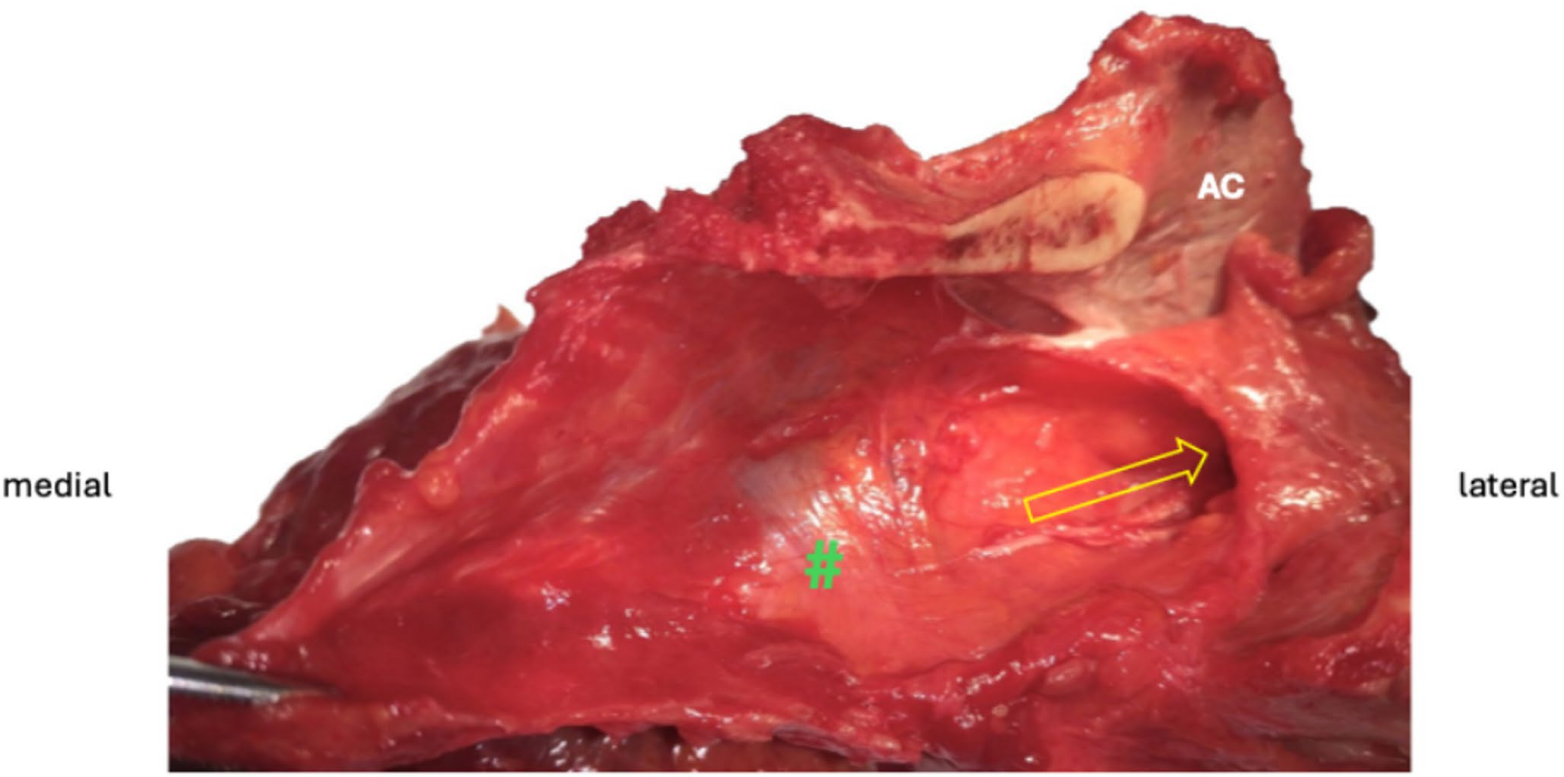
Caudodorsal view, fresh specimen. After removal of the shoulder from its anatomical position in situ, the skin and subcutaneous tissue were excised. The subscapularis muscle was detached from its attachment to the supraspinatus fossa. Subsequently, the supraspinatus muscle was removed, allowing a caudodorsal view of the supraspinatus fascia (green #). Although no longer attached to the medial margin, the fascia remained connected to the acromion (AC) and demonstrated the characteristic tube (yellow arrow) for the supraspinatus muscle and its tendon

Upon lateral and medial separation of the “subtrapezius fascia” from the underlying fatty infiltration or the fascia supraspinatus, it is noticeable that the fascia supraspinatus takes a different course at the medial end of the acromion than the subtrapezius fascia. The latter runs above the acromion and follows the trapezius to its attachment to the cranial surface of the acromion, while the supraspinatus fascia runs below the acromion to attach to its caudal surface (Fig. 3).

We also observed an additional connection between the supraspinatus fascia and the fascia of the rhomboid minor and levator scapulae muscle (Fig. 1c). However, this connection was only evident between the **superficial** layer of the supraspinatus fascia and the levator –rhomboid minor fascia, and not in its **deep** layer. In all dissected shoulders, the **superficial** layer continued laterally at the lateral of the supraspinatus fossa into the fasciae of the rhomboid minor and levator scapulae muscles.

#### Fascial channel

When combining all the attachments of the fascia together, the supraspinatus fascia forms a channel for the supraspinatus muscle (Fig. 3).

These fascial parts forming a tube for the muscle were evident in all dissected specimens, but their extent was strongly correlated with the fascia´s visible fibre content. Nine out of 16 specimens showed prominent macroscopic fibres and surrounded the caudal part of the supraspinatus muscle up to one centimetre along the bony fossa. Four out of 16 specimens only showed very thin or no fibres, and their fascial tube.

#### Vessels/nerves penetrating the supraspinatus fascia

**Fig. 4:**
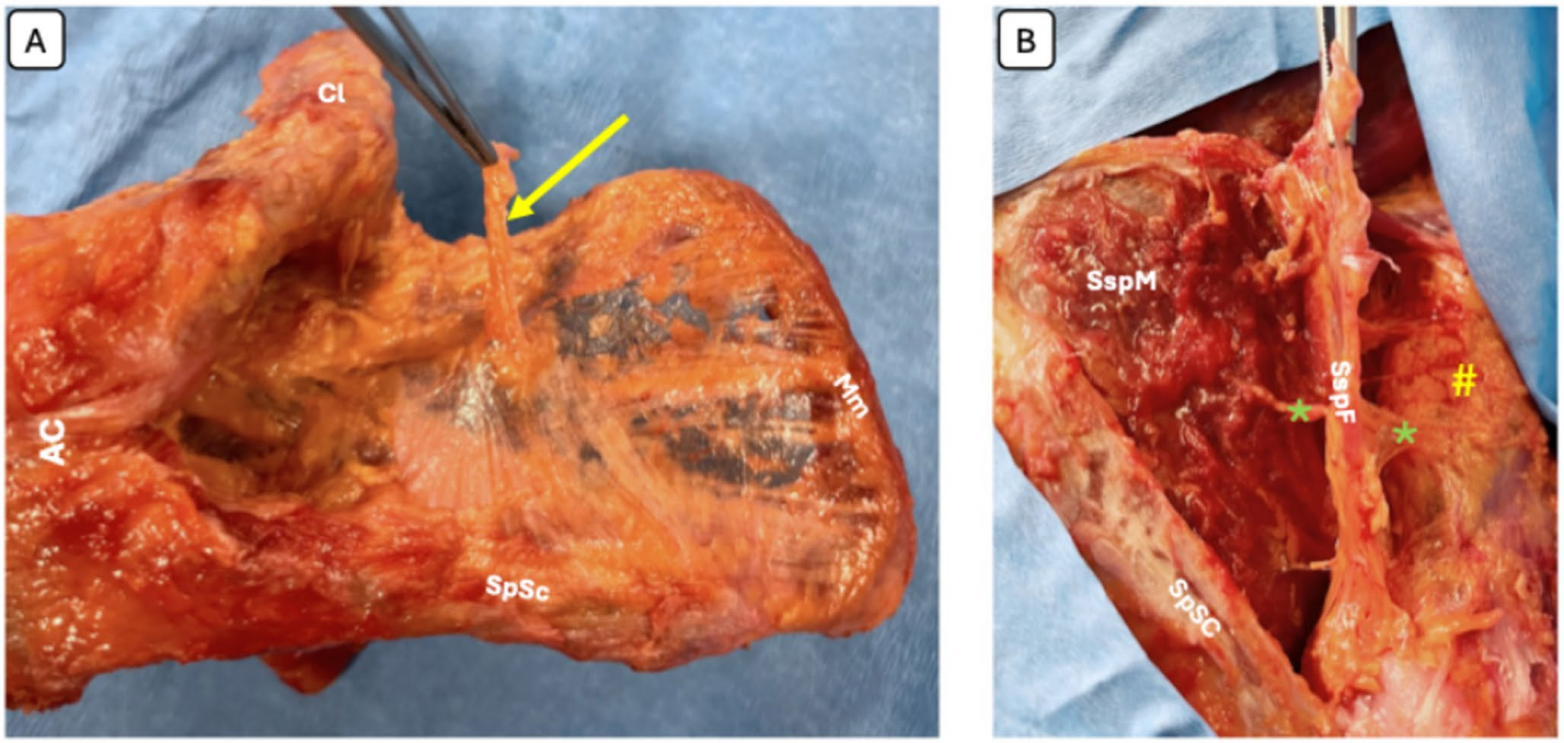
**a** Dorsal view, fresh specimen. After removal of the shoulder from its anatomical position in situ, the skin and subcutaneous tissue were removed. All muscles attached to the scapula (i.e. trapezius, deltoid, infraspinatus, subscapularis, rhomboid minor and major, levator scapulae, and supraspinatus) were removed, leaving only the supraspinatus fascia attached to its origins at the spine of the scapula (SpSc), the medial margin (Mm), the clavicle (Cl), and the acromion (Ac). This specimen shows dense white fibres within the two layers of the fascia, running in different directions. The yellow arrow indicates a fat-covered neurovascular bundle that was observed penetrating the fascia in a few cases. **b** Dorsal view, fresh specimen. After removal of skin and subcutaneous tissue, the trapezius and its underlying connective tissue (yellow #) were reflected cranially. The supraspinatus fascia (Sspf), attached to the spine (SpSc) and medial margin (Mm), was detached from these origins and reflected craniolaterally to expose the supraspinatus muscle (SspM). Green * marks several blood vessels penetrating the fascia (Sspf) from the connective tissue beneath the trapezius

While dissecting all the specimens, we discovered that four fasciae had openings within the fascia itself allowing for nerves and vessels to pass through the fascia (Fig. 4a and b), which were filled with a high content of fat. In three of those four cases, the opening in the fascia was on the lateral part of the fascia right before it ran under the acromion. One fascia, though, showed the opening right in the middle of the supraspinatus fossa (Fig. 4a).

### Histological findings

**Fig. 5:**
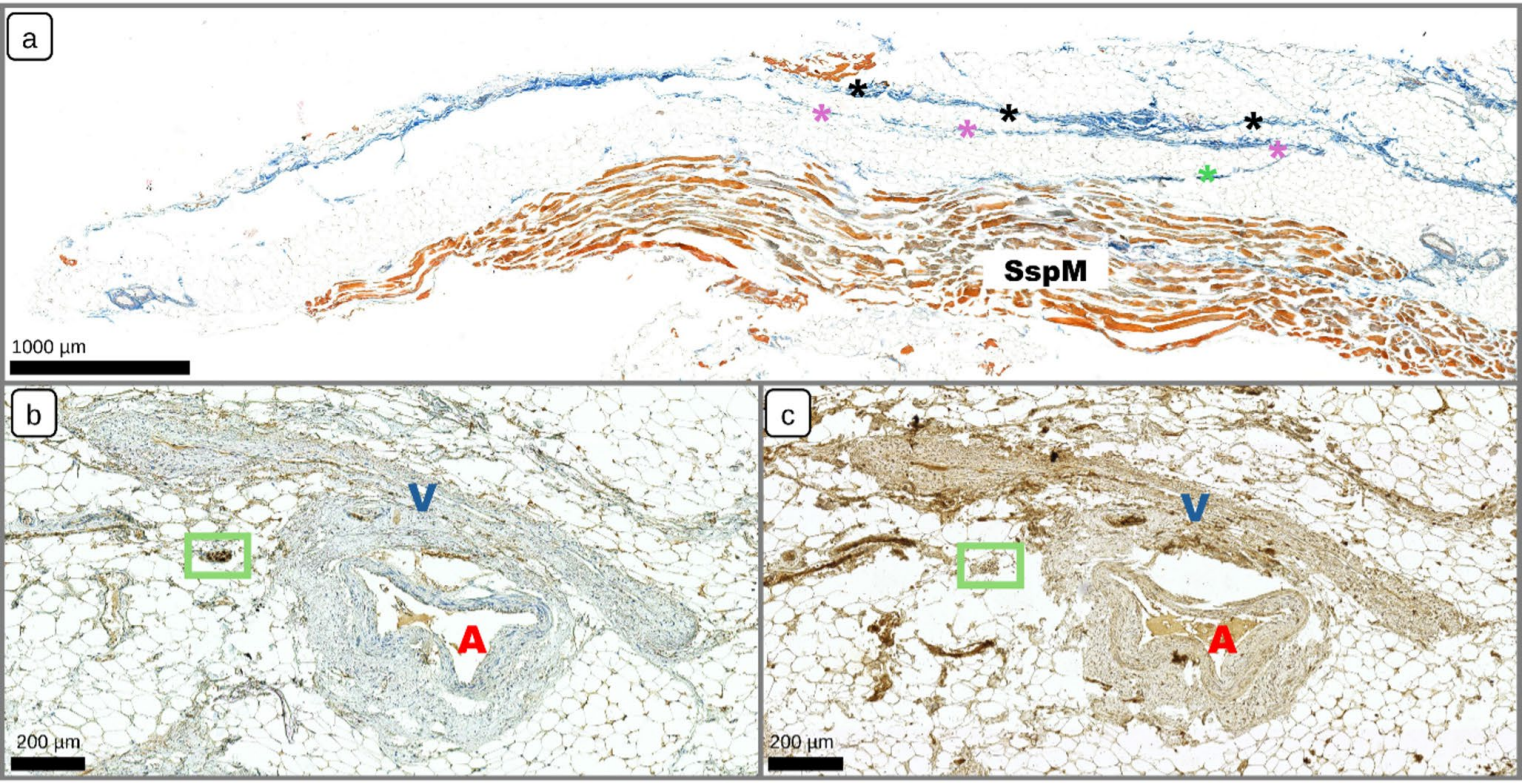
**a** Scanned K.A.O.-staining, 10×. Histological view of the supraspinatus fascia and its connection to the supraspinatus muscle (SspM). The black * marks the **superficial** layer of the supraspinatus fascia, whereas the purple * marks the **deep** layer beneath it. A distinct connection between the **deep** layer and the supraspinatus muscle (SspM) is also visible (green*). The blue staining of the layers indicates that they consist predominantly of collagen. **b** Scanned Neurofilament immunoreaction, 10×. Histological view of the supraspinatus fascia and the vessels and nerves within the fascial layers, specifically a neurovascular bundle consisting of an artery (A), a vein (V), and a nerve (green box), which exhibits dark brown precipitation. **c** Scanned Substance P immunohistochemical staining, 10×. Histological view of the same specimen shown in (b). The nerve identified in (b) demonstrated positive immunoreactivity for substance P, as indicated by the dark brown precipitation (green box)

Our macroscopic findings are supported by comparable preliminary histological findings. K.A.O. -staining showed the **superficial** and **deep** layer of the supraspinatus fascia, both of which contained collagen fibres (Fig. 5a). Immunohistochemical staining demonstrated the presence of nerves, showing positive immunoreactivity for neurofilament proteins (Fig. 5b). In addition, a high density of blood vessels was observed within the fascia (Fig. 5b and c) as well as penetrating it. Specimens stained for substance P were positive, indicating the presence of nociceptive nerve fibres within the supraspinatus fascia (Fig. 5c).

## Discussion

Our research has revealed that the connective tissue covering the supraspinatus muscle is significantly more complex than commonly described in anatomy textbooks. During the observation of twenty-four fresh body donors, we have consistently observed that the supraspinatus fascia consists of two distinct layers. This finding confirms an observation made by Singer in 1935, which he noted only in passing in his book on fasciae of the human body [15]. 

We have focused particularly on these two layers because we hypothesize that their specific anatomy—including their location, arrangement, and tissue composition—may play a role in the normal biomechanics of the shoulder or, conversely, contribute to the aetiology of pain in the scapular region. Such shoulder pain represents one of the most common musculoskeletal complaints in modern society. Despite its high prevalence, the precise underlying cause often remains unclear in many patients [13].

Although shoulder pain is highly prevalent across all segments of the population, studies indicate that its prevalence increases further in older individuals [7]. As our anatomical dissections and histological examinations are routinely performed on elderly body donors, this demographic distribution may represent a potential source of sampling bias that should be considered when interpreting our findings. Observations derived predominantly from an older population complicate the definition of a generalizable “normal” fascial architecture. Age-related structural alterations may obscure baseline morphological characteristics. To more accurately delineate typical fascial structure, investigations in younger cohorts would therefore be desirable. Unfortunately, non-invasive methods such as sonography and radiological imaging lack the precision of direct dissection and thus do not permit reliable differentiation of specific tissue layers involved. If we can clearly characterize the normal structure of these two layers of the supraspinatus fascia—as we aim to do in this work—it becomes possible to infer functional implications from form (structure-function relationships) and, in turn, to identify potential structural abnormalities that underlie common shoulder dysfunction and pain.

Firstly, the supraspinatus fascia cannot be described as simplistic. The supraspinatus fascia is often described as one layer of connective tissue with a superficial and a deep surface [16]. However, our dissections and histological testing made it evident that the supraspinatus fascia consists of at least two distinct fascial layers (Figs. 1d and 5a), a **deep** and **superficial** layer. Both layers exhibited differently oriented collagen bundles (Figs. 1a, c and d and 4a). These layers can be separated easily, often divided by fat (Fig. 1b and c) which is recognisable in all specimens when following the fascia’s course anterolaterally in direction of the clavicle.

The thickness of this fascia when combining both the **deep** and **superficial** layer, measured at three standardized points (Fig. 1a), showed an average thickness of 0.284 mm (Table 2)—thinner than Stecco’s [16] 0.7 mm median but aligning closely with Arrillaga et al. [1] at the corresponding central point (our 0.466 mm vs. their 0.52 mm). Medial thickness exceeded lateral in most specimens, driven by denser macroscopic collagen fibres medially (Fig. 1c and d). Latter being most prominent in the most medial third and above the most medial third of the spine of the scapula in all dissected specimens, while the direction of the fibres varied greatly. While some specimens showed macroscopically prominent fibres (Figs. 1c and d and 4a) others appeared clear, almost see-through (Fig. 1a and b). Nearly all specimens with less fibres medially, measured thicker laterally than they measured on the fascia´s medial part (Table 2). This contradicts Gray´s and Stecco´s observations that the fascia is always thicker medially [5, 16].

Although the supraspinatus fascia as a whole completes the osteofibrous compartment of the supraspinatus muscle (Fig. 1a, b, c and d) as described by Stecco [16], only the **superficial** layer was found to be continuous with the fasciae of the levator scapulae and rhomboid minor muscles. In agreement with the work of Kulow et al. [10] we demonstrate that it is specifically the **superficial** layer of the supraspinatus fascia that is continuous with the “levator-rhomboid-minor-fascia” while the **deep** layer is not connected. These anatomical continuities suggest that force transmission between the levator scapulae–rhomboid complex and the **superficial** layer of the supraspinatus fascia may be possible. The fascial transmission route may therefore play a role in cervicoscapular pain patterns and could be worth considering therapeutically.

In general, the **superficial** layer of the fascia extends laterally from the caudal surface of the acromion to medially continuous with the “levator-rhomboid-minor-fascia”. Dorsally it is connected to the fascia infraspinatus which can be seen by the interweaving fibres of both fasciae on the spine of the scapula. Ventrally it merges with **deep** layer to either attach to the superior transverse scapular ligament or the superior margin of the scapula directly. The **superficial** fascia does not appear to have direct osseous attachments; however, it does indirectly attach via the **deep** layer of the fascia.

The fascia´s both merged layers were found to attach to the lateral end of the clavicle (Fig. 1b) in accordance with the observations reported by Birnbaum [2]. This attachment was observed almost consistently in our specimens, as was the continuity between the supraspinatus fascia and the coracoacromial ligament. 11 of 16 also exhibited an attachment to the superior transverse scapular ligament. Therefore, a correlation may exist between this anatomical variability and the prevalence of suprascapular nerve entrapment syndrome. While Duparc et al. [3] attributed this condition primarily to the marked anatomical variability of suprascapular nerve`s relation to the superior transverse scapular ligament, our investigations demonstrated anatomical variability in the connection between the supraspinatus fascia and the superior transverse scapular ligament, which may therefore also have implications for the aetiology of nerve entrapment syndrome in this region and could be taken into account in both diagnostic and surgical contexts. Also, more recent anatomical research by Wang et al. and Heins et al. [6, 20] further underline the considerable anatomical variability of the suprascapular region and its importance for suprascapular nerve release. Heins et al. reported that ossification of the superior transverse scapular ligament was associated with significantly smaller suprascapular notch dimensions, supporting its potential role as a predisposing factor for suprascapular nerve entrapment. However, it would be of interest to investigate whether ossification of the superior transverse scapular ligament also affects the morphology of the supraspinatus fascia, including its thickness, thereby potentially contributing to the dimensions of the suprascapular notch. Furthermore, Wang et al. described considerable variability in the branching pattern of the suprascapular nerve in relation to the superior transverse scapular ligament. Future studies could therefore extend these findings by examining whether the branching pattern of the nerve is associated with the attachment pattern of the supraspinatus fascia to the superior transverse scapular ligament. While these studies primarily focused on osseous and neurovascular anatomy, the surrounding fascial structures received comparatively little attention. Our observations thus could extend the concept of impingement described by Duparc, Wang et al. and Heins et al. [3, 6, 20], incorporating the supraspinatus fascia as a potential contributory structure to the mechanical environment of the suprascapular nerve and should therefore be considered during anatomical assessment and surgical decompression.

While the **superficial** layer connects the supraspinatus fascia to other surrounding fascial systems, the **deep** layer of the fascia mainly acts as the origin of the supraspinatus muscle.

To understand the role of the fascia´s **deep** layer it is important to know that the supraspinatus muscle itself consists of two parts, an anterior and a posterior part. Vahlensieck et al. states that the posterior portion of the supraspinatus muscle is longer and attaches “to the posterior aspect of the supraspinatus fossa and the spine of the scapula” [18]. While he does not name it, the posterior part of the supraspinatus fossa is covered by the **deep** layer of supraspinatus fascia and therefore origin for the posterior portion of the supraspinatus muscle. This medial part of the **deep** layer therefore serves as the muscle attachment of the more dorsally located muscle part providing enhanced structural anchorage on that side (Fig. 2a) [18]. In contrast, our findings have found that the fascial attachments to the anterior portion of the muscle are limited and often minimal or absent (Fig. 2b). These muscle-to-fascia attachments are particularly compromised or virtually eliminated when fatty infiltration permeates the muscle belly or when a substantial fat layer accumulates superficially overlying the muscle (Fig. 2c). This differential fascial integration may contribute to the posterior portion’s primary role in shoulder abduction, while the anterior portion predominantly supports internal rotation [18], rendering the ventral region more susceptible to injury. Consequently, most rotator cuff tears preferentially involve the anterior aspect of the supraspinatus muscle [9]. One factor contributing to the regional susceptibility to injury could be the non-existent connection of the anterior muscle part to the fascia which therefore offers a weaker fascial support against mechanical stress. In chronic rotator cuff pathology, progressive fatty infiltration and associated muscle atrophy [12] could further disrupt the muscle-fascia interface, impairing overall muscular stability, mobility, and functional recovery following injury.

As Birnbaum et al. established in 1992, we can confirm with our study that the visceral surface of the subacromial bursa is connected to the lateral **deep** layer of the supraspinatus fascia [2]. Given that bursitis represents a common cause of shoulder pain, the anatomical and functional connection between the subacromial bursa and the **deep** layer of the supraspinatus fascia is of particular relevance. The connection between the fascia´s **deep** layer and the subacromial bursa may also be relevant for understanding why not all isolated bursectomies have a positive outcome. It is conceivable that fascial fibrosis may persist if the inflamed bursa involves the **deep** fascial layer [4]. As described by Langevin [11] in her work on “Fascia mobility, Proprioception, and Myofascial pain”, inflammatory changes can lead to either increased or decreased fascial mobility. In chronic states, there is a risk of fibrosis [11]. This may facilitate the transmission of inflammation to the bursa and vice versa. Therefore, our findings suggest that due to the organisation of the supraspinatus fascia in layers, inflammation may lead to adhesion between the fascial layers and a consequent reduction of general function as well as the ability of the fascia´s two layers to slide on one another.

Our histological findings show the existence of neurovascular bundles (Fig. 5b and c), with nerves tested positives for substance P (Fig. 5c), which confirms the presence of nociceptive nerve fibres within the fascia. While our histological data does not allow us to determine whether the fascia itself is directly innervated or whether these fibres merely transverse the tissue, it is evident that fascial stiffening—with subsequent impairing of neural and vascular supply—could potentially have physiological consequences. This may be similar to the findings of Wilke [22] who describe adhesion of fascia when describing pain in the lumbar fascia [22]. Therefore, in case of chronic inflammatory conditions associated with shoulder pain, potential fibrotic alterations of the fascia could be considered as a contributing factor, requiring further research.

## Limitations

This research is descriptive. Therefore, several limitations of this research should be acknowledged. We are aware that the use of older body donors for science limits the ability to fully capture interindividual variability as well as it may not reflect the situation in younger people. Post mortal changes or differences related to age, sex, physical activity, or degenerative changes could not be systematically controlled and may have influenced the fascial thickness, fibre density or muscle-fascia adhesion and therefore may have influenced our results. We are also aware that in the absence of the central nervous system and without physiological muscle activity, physiological tensions could not be evaluated. The study is descriptive in nature and therefore only allows suggestions regarding biomechanical function or clinical relevance. Although most of the dissections were performed on fresh-frozen specimens, the tissue samples used for histological analysis were formalin-fixed which may have led to alter the tissues morphology or collagen organisation and therefore impacted our results. Furthermore, histological analyses were performed in a limited number of specimens and should be considered preliminary. Interobserver reliability of the macroscopic observations was not assessed.

## Conclusion

In conclusion, our quantitative and qualitative findings refine textbook depictions of the supraspinatus fascia as a single uniform layer. We have documented the multilayer complexity, measurable variability in thickness and attachments, differential muscle adherence, and nociceptive elements. Ultimately, our findings demonstrate that the supraspinatus fascia represents a distinct multilayered anatomical system rather than a uniform connective tissue sheet. Recognition of this complexity may improve our understanding of shoulder anatomy and provides a basis for future studies investigating its biomechanical and clinical significance.

## Data Availability

No datasets were generated or analysed during the current study.
